# Supplementary material for: Cholinergic basal forebrain nucleus of Meynert regulates chronic pain-like behavior via modulation of the prelimbic cortex
Source: Nat Commun. 2022 Aug 25;13:5014. doi: 10.1038/s41467-022-32558-9 (PMC9411538; doi:10.1038/s41467-022-32558-9)
Supplement: Supplementary file 1 — Supplementary Information [file 41467_2022_32558_MOESM1_ESM.pdf]

Supplementary Information for

Cholinergic basal forebrain nucleus of Meynert regulates chronic pain-like behaviour via modulation of the prelimbic cortex

Manfred J. Oswald, Yechao Han, Han Li, Samuel Marashli, Deniz N. Oglo, Bhavya Ojha, Paul V. Naser, Zheng Gan, Rohini Kuner

Pharmacology Institute, Medical Faculty Heidelberg, Heidelberg University, Im Neuenheimer Feld 366, 69120 Heidelberg, Germany

Correspondence to: rohini.kuner@pharma.uni-heidelberg.de

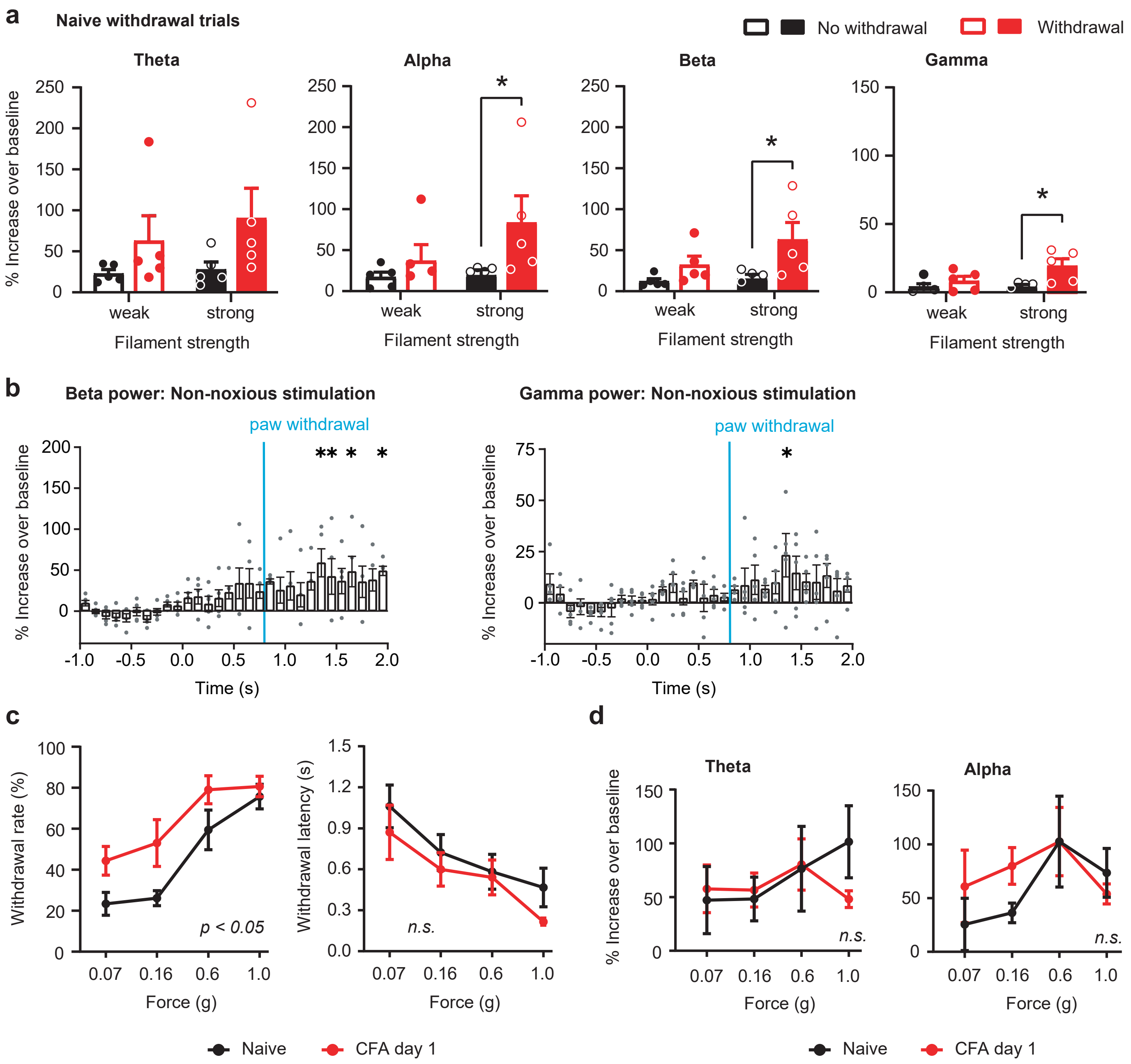

Suppl. Fig. 1: Additional data on oscillatory activity recorded in vivo in electrophysiological experiments in awake, behaving mice using tetrodes implanted in the NBM. (a) Quantification of power of oscillatory activity in frequency ranges theta (4 – 8 Hz), alpha (8 – 14 Hz), beta (14 – 30 Hz) and gamma (30 – 100 Hz) derived from time frequency representations, represented as % change in 2 s post-application period over 1 s baseline activity prior to stimulus application, in response to non-noxious (weak) and noxious (strong) mechanical von Frey stimuli in mice showing nocifensive withdrawal response as compared to no response. * $p < 0.05$ (0.0043, 0.0023, and 0.0223 for alpha, beta, and gamma, respectively), two-way ANOVA with Sidak's multiple comparisons test. **(b)** Time course of change in gamma or beta frequency oscillatory power in response to non-noxious von Frey stimulation, calculated as % increase over the mean of baseline (1s prior to stimulus application); time of paw withdrawal is indicated by vertical blue line. *$p<0.05$ (beta: 0.0007, 0.0329, 0.0094, 0.0077, from left to right; gamma: 0.0068); One-way repeated measures ANOVA with Dunnet's multiple-comparison test. **(c)** Unilateral paw injection of Complete Freund's adjuvant (CFA) induces mechanical hypersensitivity (left) without a change in reaction time (right). **(d)** Comparison of stimulus response curves showing changes in theta and alpha activity rhythms % change in 2 s post-application period over 1 s baseline activity prior to application of graded von Frey mechanical stimuli to the hindpaw between naïve and inflamed states. (c, d) p values in inset (0.0136 in c) represent two-way ANOVA-based comparison of the two entire stimulus-response curves; n = 5 mice/group in panels a to d. Data are presented as mean +/- standard error of the mean (S.E.M.).

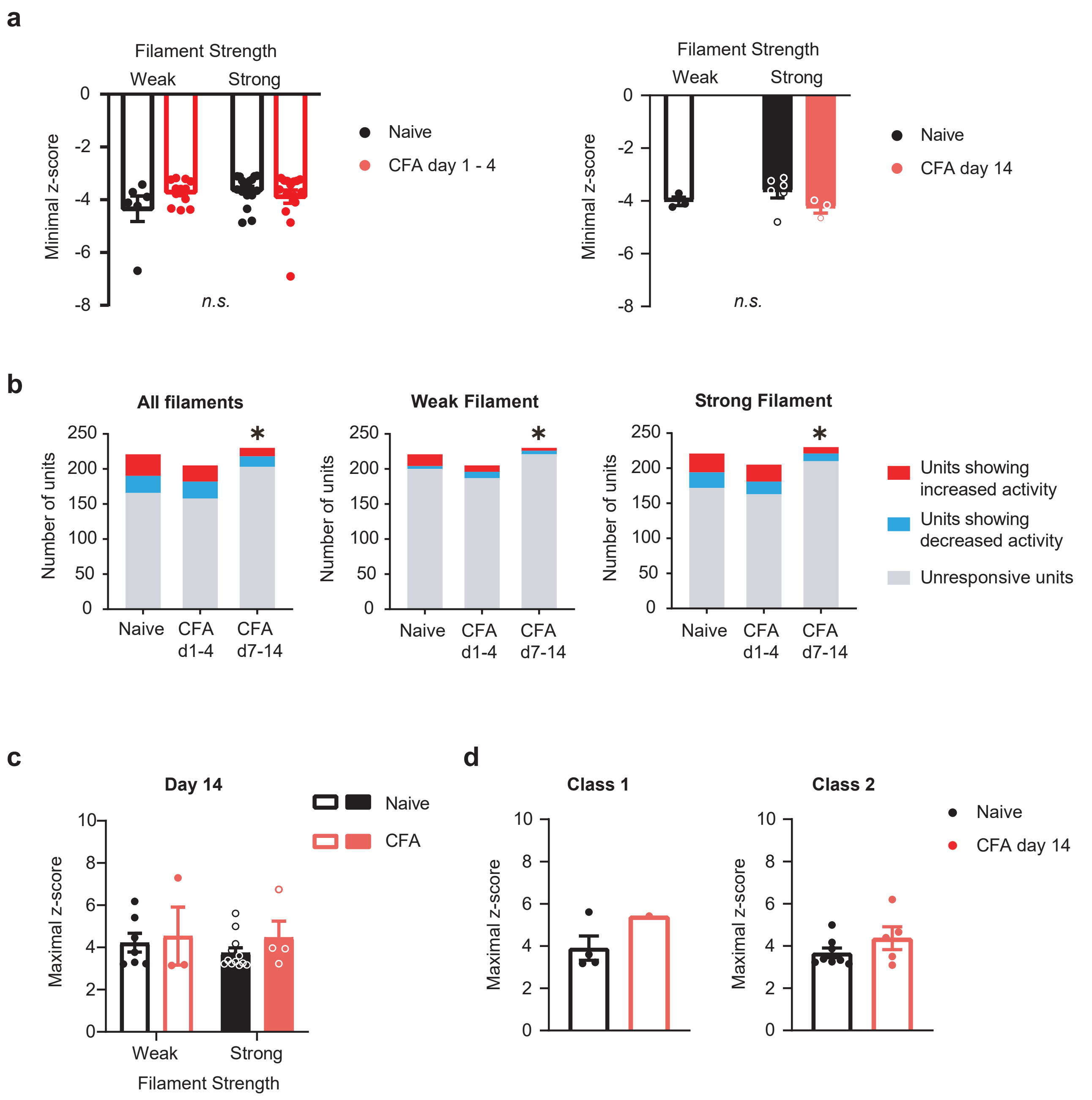

Suppl. Fig. 2: Additional data on resolving changes in NBM activity during acute nociception and inflammatory at the single cell level at late stages after intraplantar hindpaw CFA injection when nociceptive sensitivity is back to normal. **(a)** Distribution of NBM units responding to mechanical stimulation in naïve (sham) conditions and during hindpaw CFA-induced peak inflammatory pain (day 1-4) and after inflammatory pain has largely abated (day 9-14). * $p < 0.05$ (all: 0.0038, weak: 0.0159, strong: 0.0013), Chi-square analysis. **(b)** The maximum reduction of activity over average baseline values (Z-score) for units inhibited by mechanical stimulation is demonstrated in naive mice and different periods post-CFA. **(c, d)** The maximum increase of activity over average baseline values (Z-score) for units excited by mechanical stimulation is demonstrated in naive mice and after CFA-induced inflammatory pain is over (day 14). In panel d, units are subdivided into class 1 and class 2 (fast spiking) types of neurons based on waveform. n = 5 mice/group. Two-way ANOVA (b, c) and unpaired two-sided t-tests (d). Data are presented as mean +/- standard error of the mean (S.E.M.).

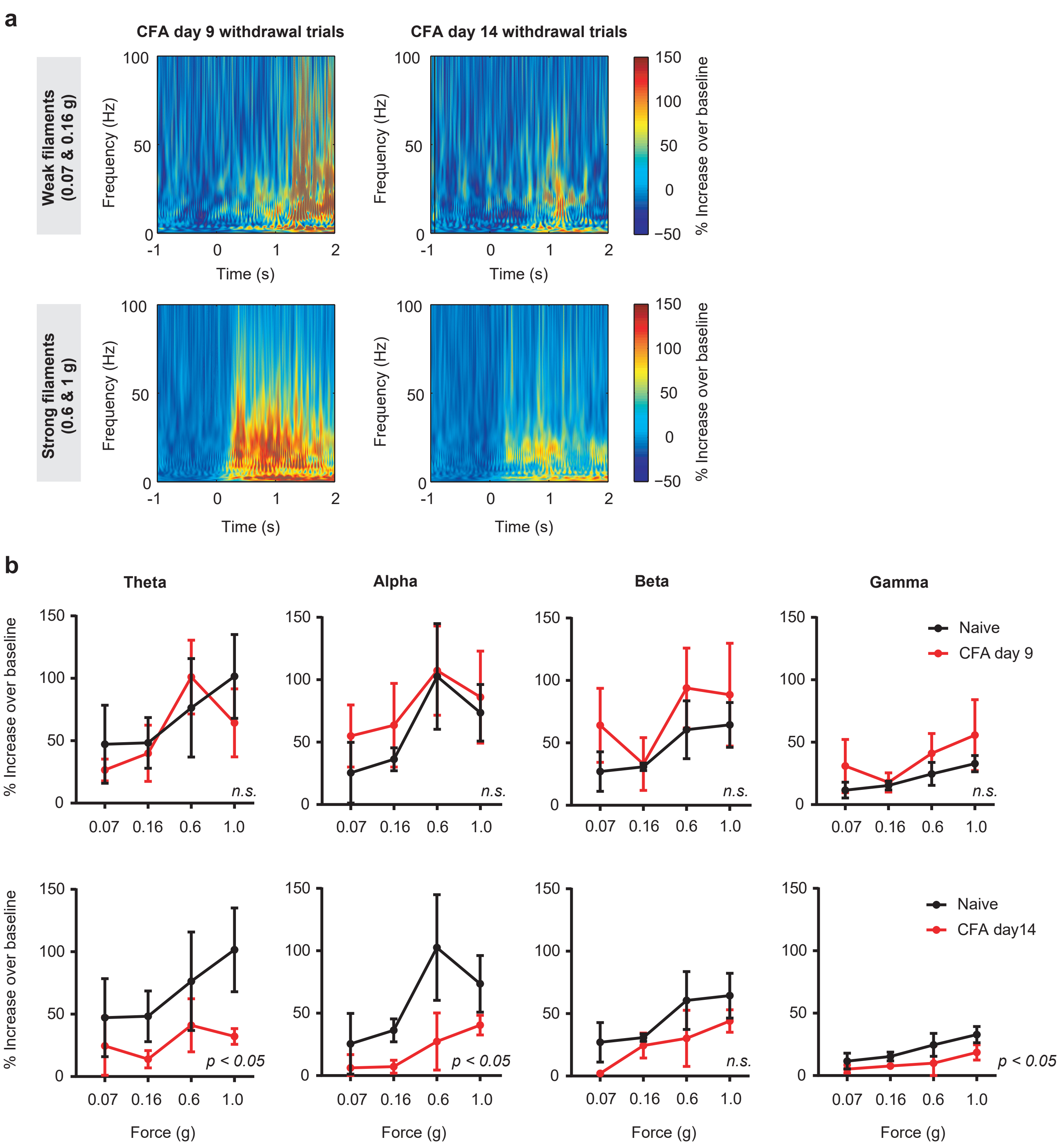

Suppl. Fig. 3: Analysis of oscillatory rhythmic activity in the NBM at late stages after intraplantar hindpaw CFA injection when nociceptive sensitivity is back to normal. **(a)** Time frequency representation of spectral modulation in the NBM for all trials with paw withdrawal response to either low intensity forces (0.07 g and 0.16 g; weak filaments) or forces close to or at nociceptive threshold (0.6 g and 1 g; strong filaments) at days 9 and 14 post-CFA. **(b)** Comparison of stimulus response curves showing changes in theta and alpha activity rhythms % change in 2 s post-application period over 1 s baseline activity prior to application of graded von Frey mechanical stimuli to the hindpaw between naïve and inflamed states. p values in inset (theta, 0.0314; alpha, 0.0148; gamma, 0.0466) represent two-way ANOVA-based comparison of the two entire stimulus-response curves. n = 5 mice/group. * $p < 0.05$, two-way ANOVA with Sidak's multiple comparisons test. Data are presented as mean +/- standard error of the mean (S.E.M.).

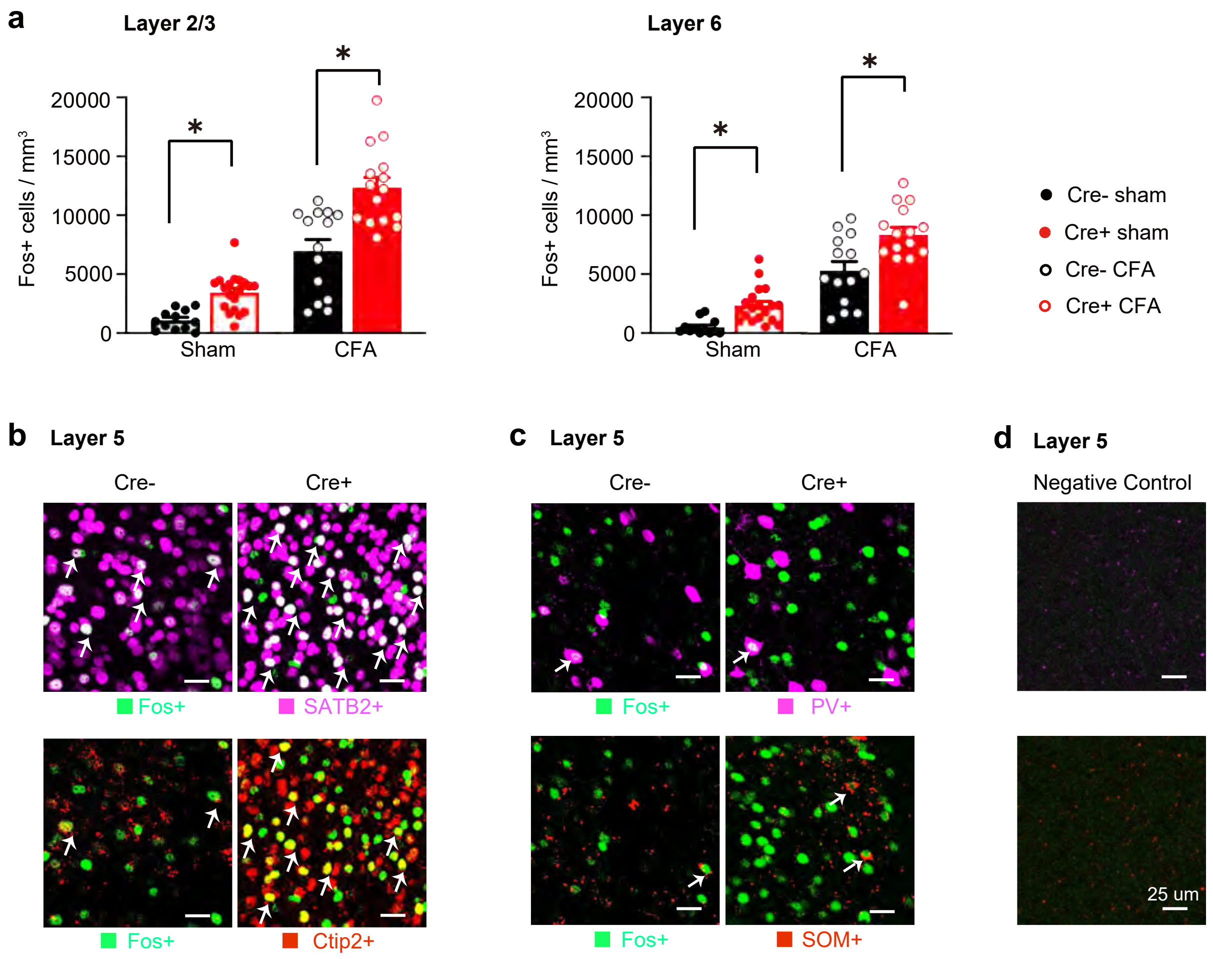

Suppl. Fig. 4: Typical examples and additional controls for immunohistochemical analysis of identity of prelimbic (PL) neurons. (a) Quantification of activity marker Fos across layers 2/3 or layer 6 of PL (**panel a**) and typical examples of Fos co-labelling with sub-classes of excitatory projection neuron classes (SATB2-expressing or Ctip2-expressing; **panel b**) and inhibitory neurons (Parvalbumin-expressing or Somatostatin-expressing; **panel c**) in response to blue light stimulation of NBM-PL cholinergic-GABAergic projections (represented by Cre+ mice) or control (represented by Cre- mice). N = 5 mice/group for panels a-c; *p<0.05 (layer 2/3: sham 0.0338, CFA < 0.0001; layer 6: sham 0.0390, CFA 0.0005), two-way ANOVA followed by post-hoc Sidak's test for panel a. Scale bar = 25 microns. **(d)** Negative controls lacking primary antibodies. Data are presented as mean +/- standard error of the mean (S.E.M.).

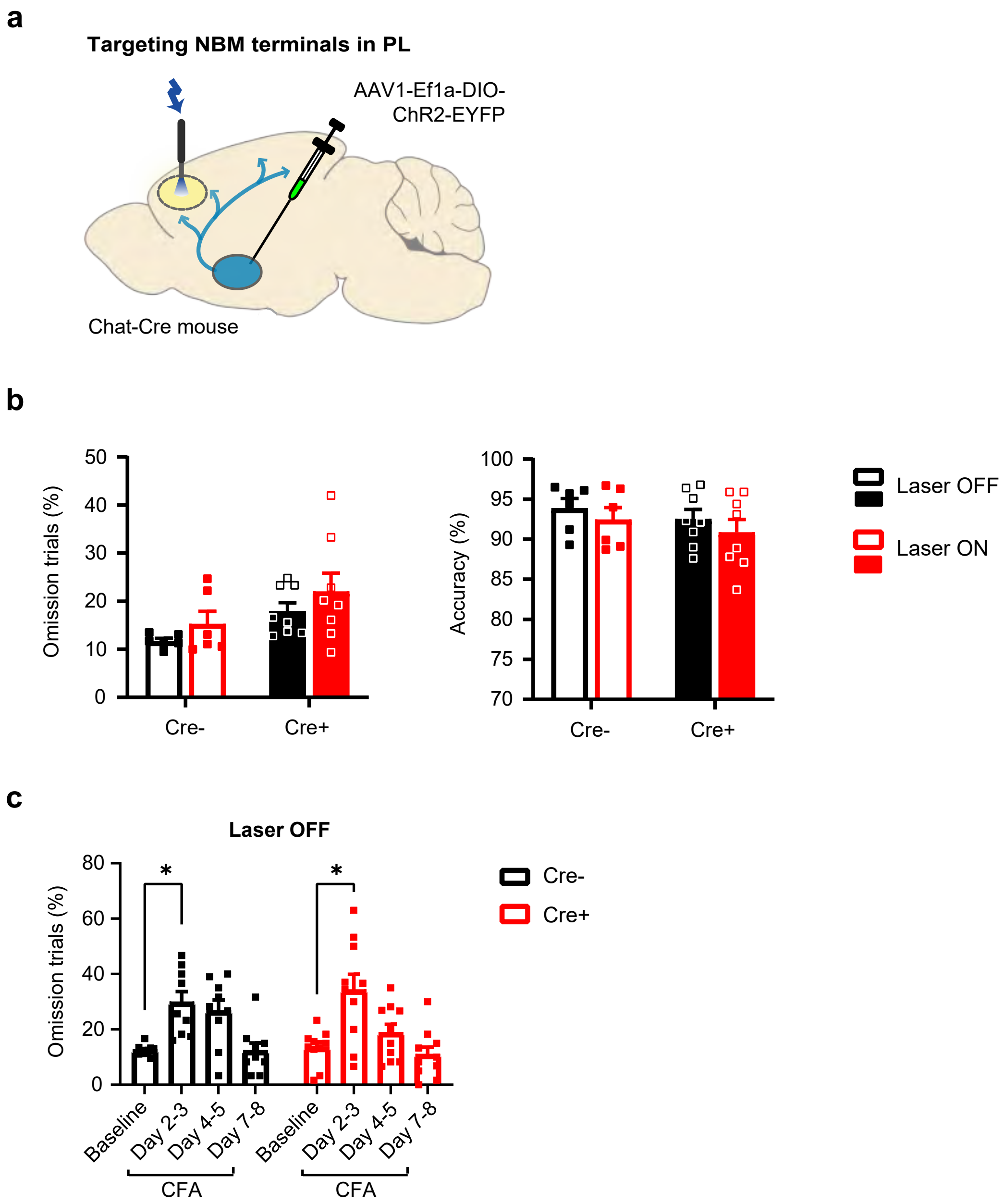

Suppl. Fig. 5: Additional controls for attention-related experiments. (a, b) In mice with optogenetic stimulation of NBM cholinergic-GABAergic projections to the PL with blue laser light **(a)**, attention-related parameters are not different between trials with laser-induced stimulation and without laser **(b)**. N = 6 ChAT-Cre- and 8 ChAT-Cre+ mice; two-way ANOVA test. **(c)** Laser-OFF controls for experiments addressing potential alterations in attentional behavior upon induction of inflammatory pain following hindpaw CFA injection as compared to baseline behavior in ChAT-Cre+ mice (red bars) and Cre- control mice (black bars). CFA-induced attentional deficits occur independently of activity of NBM-PL cholinergic projections. N = 9 ChAT-Cre- and 10 ChAT-Cre+ mice; * $p < 0.05$ (Cre- 0.0105, Cre+ 0.0059), two-way ANOVA with Sidak's multiple comparisons test. Data are presented as mean +/- standard error of the mean (S.E.M.).
