# Supplementary material for: Cholinergic basal forebrain nucleus of Meynert regulates chronic pain-like behavior via modulation of the prelimbic cortex
Source: Nat Commun. 2022 Aug 25;13:5014. doi: 10.1038/s41467-022-32558-9 (PMC9411538; doi:10.1038/s41467-022-32558-9)
Supplement: Supplementary file 3 — Reporting Summary [file 41467_2022_32558_MOESM3_ESM.pdf]

nature portfolio

Corresponding author(s): Prof. Dr. Rohini Kuner

Last updated by author(s): Jul 9, 2022

Reporting Summary

Nature Portfolio wishes to improve the reproducibility of the work that we publish. This form provides structure for consistency and transparency in reporting. For further information on Nature Portfolio policies, see our Editorial Policies and the Editorial Policy Checklist.

Statistics

For all statistical analyses, confirm that the following items are present in the figure legend, table legend, main text, or Methods section.

n/a	Confirmed	
☐	☒	The exact sample size (*n*) for each experimental group/condition, given as a discrete number and unit of measurement
☐	☒	A statement on whether measurements were taken from distinct samples or whether the same sample was measured repeatedly
☐	☒	The statistical test(s) used AND whether they are one- or two-sided<br>*Only common tests should be described solely by name; describe more complex techniques in the Methods section.*
☒	☐	A description of all covariates tested
☐	☒	A description of any assumptions or corrections, such as tests of normality and adjustment for multiple comparisons
☐	☒	A full description of the statistical parameters including central tendency (e.g. means) or other basic estimates (e.g. regression coefficient) AND variation (e.g. standard deviation) or associated estimates of uncertainty (e.g. confidence intervals)
☐	☒	For null hypothesis testing, the test statistic (e.g. *F*, *t*, *r*) with confidence intervals, effect sizes, degrees of freedom and *P* value noted<br>*Give P values as exact values whenever suitable.*
☒	☐	For Bayesian analysis, information on the choice of priors and Markov chain Monte Carlo settings
☒	☐	For hierarchical and complex designs, identification of the appropriate level for tests and full reporting of outcomes
☒	☐	Estimates of effect sizes (e.g. Cohen's *d*, Pearson's *r*), indicating how they were calculated

Our web collection on statistics for biologists contains articles on many of the points above.

Software and code

Policy information about availability of computer code

Data collection	Cheetah data acquisition softare (Neuralynx, version 5.6.3); Any-Maze video tracking software (Stoelting Co., version 6.06); ABET II TOUCH software (Lafayette Instruments, version 2.15); Leica Application Suite X (LAS X, version 3.3.0).
Data analysis	Matlab analysis scripts associated with the manuscript are deposited along with the electrophysiology dataset in a repository (heiDATA, 2022) with accession code, DOI: 10.11588/data/ET9G9X.

For manuscripts utilizing custom algorithms or software that are central to the research but not yet described in published literature, software must be made available to editors and reviewers. We strongly encourage code deposition in a community repository (e.g. GitHub). See the Nature Portfolio guidelines for submitting code & software for further information.

Data

Policy information about availability of data

All manuscripts must include a data availability statement. This statement should provide the following information, where applicable:

- Accession codes, unique identifiers, or web links for publicly available datasets
- A description of any restrictions on data availability
- For clinical datasets or third party data, please ensure that the statement adheres to our policy

The electrophysiology data generated in this study have been deposited in the heiDATA repository under accession code [https://doi.org/10.11588/data/ET9G9X]. Source data are provided with this paper.

Field-specific reporting

Please select the one below that is the best fit for your research. If you are not sure, read the appropriate sections before making your selection.

- [x] Life sciences
- [ ] Behavioural & social sciences
- [ ] Ecological, evolutionary & environmental sciences

For a reference copy of the document with all sections, see nature.com/documents/nr-reporting-summary-flat.pdf

Life sciences study design

All studies must disclose on these points even when the disclosure is negative.

Sample size	Our sample sizes are similar to those reported in previous publications. Based on previous studies we have determined the sample size using G-power analyses and therefore have a very clear set of what sample size is required for the behavioral and histochemical data reported.
Data exclusions	For the analysis of evoked single unit activity, recording sessions with fewer than 3 withdrawal trials as well as units with a mean firing rate < 1 Hz were excluded from this analysis. For testing attention behaviour with the 5-CSRT task, animals that did not achieve a baseline criterion of fewer than 30 % omission trials were excluded from the experiment.
Replication	All experiments were successfully replicated at least once with several animals. The precise animal numbers are given in the figure legends.
Randomization	Groups were randomized and mice were allocated to experimental groups by a researcher different from the experimentor
Blinding	Experimenters were always blinded to the identity of the treatment groups.

Reporting for specific materials, systems and methods

We require information from authors about some types of materials, experimental systems and methods used in many studies. Here, indicate whether each material, system or method listed is relevant to your study. If you are not sure if a list item applies to your research, read the appropriate section before selecting a response.

Materials & experimental systems

n/a	Involved in the study	
[ ]	[x]	Antibodies
[x]	[ ]	Eukaryotic cell lines
[x]	[ ]	Palaeontology and archaeology
[ ]	[x]	Animals and other organisms
[x]	[ ]	Human research participants
[x]	[ ]	Clinical data
[x]	[ ]	Dual use research of concern

Methods

n/a	Involved in the study	
[x]	[ ]	ChIP-seq
[x]	[ ]	Flow cytometry
[x]	[ ]	MRI-based neuroimaging

Antibodies

Antibodies used

The following polyclonal antibodies were used: Rabbit anti-Fos (ab190289, Abcam, 1:1000), affinity-purified goat anti-ChAT (AB144P, Merck, 1:250), chicken anti-GFP (ab13970, Abcam, 1:1000), rat anti-somatostatin (MAB354, EMD Millipore, 1:300), guinea pig anti-parvalbumin (GP72, Swant, 1:5000), rat anti-Ctip2 (ab18465, Abcam, 1:500), and guinea pig anti-SATB2 (327004, Synaptic Systems, 1:200).

Donkey anti-Rabbit IgG (H+L), cross-adsorbed, Alexa 488 conjugated (Cat # A32790)
Donkey anti-Rabbit IgG (H+L), cross-adsorbed, Alexa 594 conjugated (Cat # A32754)
Donkey anti-Goat IgG (H+L), cross-adsorbed, Alexa 633 conjugated (Cat # A-21082)
Donkey anti-Goat IgG (H+L), cross-adsorbed, Alexa 488 conjugated (Dat # A-11055)
Donkey anti-Rat IgG (H+L), cross-adsorbed, Alexa 594 conjugated (Cat # A48271)
Goat anti-Chicken IgY (H+L), cross-adsorbed, Alexa 488 conjugated (Cat # A32931)
Goat anti-Guinea pig IgG (H+L), cross-adsorbed, Alexa 647 conjugated (Cat # A-21450)

All of these above secondary antibodies were purchased from Thermo Fisher Scientific.

Validation

All the primary antibodies were used in non-living tissue for immunohistochemistry (IHC). These antibodies are extensively used for IHC purpose by the scientific community with numerous species-relevant citations for each primary antibody available on the manufacturers website. Except for the anti-GFP antibody that is species-independent, all manufacturers specifically state reactivity with mouse. We routinely performed negative controls by omitting primary antibodies and show negative image examples for Fos, Ctip-2, SATB2, parvalbumin, and somatostatin in Supplmentary Figure 4D.

Animals and other organisms

Policy information about studies involving animals; ARRIVE guidelines recommended for reporting animal research

Laboratory animals	Adult (8-34 weeks) C57BL/6J male/female mice (25 - 30 g) of wild-type, Chat-IRES-Cre mice (B6;129S6-Chattm2(cre)Lowl//Uhg) with a C57BL/6 background, and Rbp4-Cre mice (B6.FVB/CD1-Tg(Rbp4cre)KL100Gsat/Uhg) with a C57BL/6 background were used in this study. Mice were housed in groups of 2–3 per cage (in ventilation unit) with food and water ad libitum on a 12 h light / 12 h dark cycle. Room temparature and humidity were ranging from 20-23 °C and 40-60%.
Wild animals	The study did not involve wild animals.
Field-collected samples	No field collected samples were used this study.
Ethics oversight	All of the animal experiments were conducted according to the ethical guidelines of 'Protection of Animals Act' under supervision of the 'Animal Welfare Officers' of Heidelberg University and were approved by the local governing body named 'Regierungspräsidium Karlsruhe: Abteilung 3 - Landwirtschaft, Ländlicher Raum, Veterinär- und Lebensmittelwesen', Germany (Approval numbers: G-44/17 and G-184/18). ARRIVE guidelines were followed.

Note that full information on the approval of the study protocol must also be provided in the manuscript.
